# Equilibrium Isotherm, Kinetic Modeling, Optimization, and Characterization Studies of Cadmium Adsorption by Surface-Engineered *Escherichia coli*

**DOI:** 10.18869/acadpub.ibj.21.6.380

**Published:** 2017-11

**Authors:** Vida Tafakori, Reza Zadmard, Fatemeh Tabandeh, Mohammad Ali Amoozegar, Gholamreza Ahmadian

**Affiliations:** 1 Department of Cell and Molecular Sciences, School of Biological Science, Kharazmi University, Tehran, Iran; 2 Department of Industrial and Environmental Biotechnology, National Institute of Genetic Engineering and Biotechnology (NIGEB), Tehran, Iran; 3 Chemistry and Chemical Engineering Research Center of Iran, Tehran, Iran; 4 Extremophiles Laboratory, Department of Microbiology, School of Biology, College of Science, University of Tehran, Tehran, Iran

**Keywords:** Adsorption, Kinetics, Response surface methodology, Fourier transform infrared spectrophotometer

## Abstract

**Background:**

Amongst the methods that remove heavy metals from environment, biosorption approaches have received increased attention because of their environmentally friendly and cost-effective feature, as well as their superior performances.

**Methods:**

In the present study, we investigated the ability of a surface-engineered *Escherichia coli*, carrying the cyanobacterial metallothionein on the cell surface, in the removal of Ca (II) from solution under different experimental conditions. The biosorption process was optimized using central composite design. In parallel, the kinetics of metal biosorption was studied, and the rate constants of different kinetic models were calculated.

**Results:**

Cadmium biosorption is followed by the second-order kinetics. Freundlich and Langmuir equations were used to analyze sorption data; characteristic parameters were determined for each adsorption isotherm. The biosorption process was optimized using the central composite design. The optimal cadmium sorption capacity (284.69 nmol/mg biomass) was obtained at 40°C (pH 8) and a biomass dosage of 10 mg. The influence of two elutants, EDTA and CaCl_2_, was also assessed on metal recovery. Approximately, 68.58% and 56.54% of the adsorbed cadmium were removed by EDTA and CaCl_2_ during desorption, respectively. The Fourier transform infrared spectrophotometer (FTIR) analysis indicated that carboxyl, amino, phosphoryl, thiol, and hydroxyl are the main chemical groups involved in the cadmium bioadsorption process.

**Conclusion:**

Results from this study implied that chemical adsorption on the heterogeneous surface of *E. coli* E and optimization of adsorption parameters provides a highly efficient bioadsorbent.

## INTRODUCTION

Cadmium contamination has serious potential implications for soil, water, and human health[1]. Unlike organic pollutants, heavy metals are not degradable and may persist for relatively long periods of time in aquatic and terrestrial environments[2]. Amongst the methods that remove heavy metals from environment, biosorption approaches have received increased attention because of their environmentally friendly and cost-effective feature, as well as their superior performances. Due to these properties, the biosorption is usually preferred to physical or chemical remediation technologies. Biosorption takes advantage of naturally occurring biomaterials for heavy metal removal[3]. Biomaterials such as bacteria, yeasts, algae, and some plants have been successfully applied for pollutant adsorption, and it has been shown that these materials demonstrate a high capacity in removing large amounts of heavy metals from wastewaters. Other benefits of using biomaterials include lower cost of investment, lower energy consumption, and high removal efficiency of the heavy metals[4]. However, natural biomaterials have neither a high selectivity nor a high capacity for the adsorption of heavy metals. Surface-engineered bacteria solve these problems by expressing the large amounts of selective sorbent motifs, such as metallothioneins (MTs), on their surfaces. MTs are low-molecular-weight (6–7 kDa), cysteine-rich, metal-binding proteins found in animals, higher plants, eukaryotic microorganisms, and some prokaryotes[5]. MTs have the ability to bind to both physiological (such as zinc, copper, and selenium) and xenobiotic (such as cadmium, mercury, silver, and arsenic) heavy metals through the thiol group of its cysteine residues, which represents nearly the 30% of its amino acidic residues[6].

The main advantages associated with the surface expression of metal adsorbents, in comparison to intracellular expression, include the elimination of the time-consuming and rate-limiting step of crossing the membrane, prevention of interference with redox pathways in the cytosol, the uptake of any heavy metal of interest by expressing its binding peptide on the surface of cell, and recycling of the biosorbents[7-11]. Various bacterial-based systems have been developed for heavy metal removal by using microorganisms over-expressing MTs on their surfaces[9,12]. Surface-engineered bacteria show enhanced adsorption of heavy metals and offer a promising strategy with respect to the development of bacterial-based biosorbents for the removal of heavy metal ions from wastewater. Cadmium is considered as one of the most toxic heavy metals, and in fact, cyanobacterial MTs such as SmtA have been shown to have high affinity for Cd (II), Zn (II), and Cu (II) ions[13]. In our previous research, the Lipoprotein-outer membrane protein A (Lpp’-OmpA) system was used for surface display of cyanobacterial MTs[14]. The ability of these MTs to be displayed on the bacterial surface has not been evaluated so far. The Lpp’-OmpA system consists of a signal sequence and the first nine N-terminal amino acids of the major *E. coli* Lpp′ joined to a transmembrane domain (residues 46 to 159) of the outer membrane protein of OmpA[15].

The purpose of this paper was to study the kinetics and equilibrium isotherm of the Cd (II) biosorption process. We optimized the uptake of Cd (II) by surface-engineered *E. coli* using the statistical design of experiments. In addition, the present study investigated the impact of desorption agents on the recovery of adsorbed Cd (II). Surface functional groups of bacterial cells involved in cadmium adsorption were also determined using Fourier transform infrared spectrophotometer analysis.

## MATERIALS AND METHODS

### Microorganism and media

The recombinant *E. coli* strain E, engineered with a cyanobacterial MT, SmtA, using the pET26b-Lpp′-OmpA expression vector was obtained from National Institute of Genetic Engineering and Biotechnology (Tehran, Iran)[14]. Luria Bertani (LB) was used as the growth medium and supplemented with kanamycin sulfate to a final concentration of 50 mg/mL. Isopropyl β-D-1-thiogalactopyranoside, as an inducer, was added to the culture medium when the cells reached an optical density of 0.6 at 600 nm. After induction period, the culture was incubated at 25ºC for 5 h. The cells were subsequently harvested by centrifugation at 4000 ×g and freeze dried until further use.

### Preparation of metal solutions

Metal solutions were prepared by diluting a 1000 mg/L stock solution of Cd (NO_3_)_2_.4H_2_O, with 0.1 M trisaminomethane-hydrochloric acid (Tris-HCl) to obtain concentrations between 10-110 mg/L. For each solution, the initial Cd (II) concentration and the concentration in the samples following the biosorption treatment process were determined using a flame atomic absorption spectrometer (Perkin Elmer Aanalyst, USA).

### Cadmium adsorption studies

Batch adsorption experiments were conducted to study kinetic models, equilibrium isotherms, and the effect of different variables on cadmium adsorption, consisting of pH, temperature, and mass dosage. Each experiment was carried out in 100-mL Erlenmeyer flasks containing 10 mL of Cd (II) solution by shaking at 100 rpm. Then biomass was separated by centrifugation at 4000 *×*g and filtered through a Whatman filter paper with a pore size of 25 µm. Filtered samples were then analyzed for residual Cd (II) ion concentration using an analyst 700 atomic adsorption spectrometer (Perkin Elmer Aanalyst 700, USA). A control experiment was also carried out using the same solution and equipment, but in the absence of the bioadsorbent, *E. coli* E. Solute uptake by the recombinant *E. coli* E strain can be calculated from the differences between the initial and final quantities of the solute contained in the supernatant as follows:


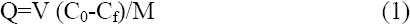


where Q is the solute uptake (mg/g); C_0_ and C_f_, the initial and equilibrium solute concentrations in solution (mg/L), respectively; V, solution volume (L); M, the mass of the biosorbent (g)[16].

### Biosorption kinetics

The sorption kinetics data provide valuable insights into the reaction pathways, the mechanism of the sorption reaction, and solute uptake[16]. The pseudo-first-order and pseudo-second-order biosorption models were applied to describe the kinetics of biosorption. The initial Cd (II) concentration was 20 mg/L in Tris-HCl buffer, pH 6.5. The sorption time varied between 5 and 100 min, and temperature was set at 30ºC. At different times, each flask was removed from the shaker, and the biomass was centrifuged as mentioned above and then filtered. Finally, the solutions were analyzed to measure the residual Cd (II) concentration. The pseudo-first order model points that the rate of adsorption sites occupation is proportional to the number of unoccupied sites[17].

The linear equation for this model is:


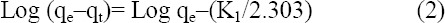


where q_e_ and q are the amounts of metal ions adsorbed at equilibrium and at any time (t), respectively (nmol/mg) onto the biosorbent surface, and K_1;_ is the rate constant of the first-order biosorption[17]. In the pseudo-second order model, it is assumed that the rate of the occupation of adsorption sites is proportional to the square of the number of unoccupied sites[17]. Linear equation for this model is:


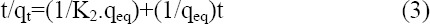


where q_eq_ and q_t_ are the amounts of metal ions adsorbed on the biosorbent at equilibrium and at any time (t), respectively (nmol/mg), and K_2_ is the rate constant of second-order biosorption (mg/nmol min)[17].

The linear regression curve fitting procedure was performed with Microsoft Excel (version 7). The goodness of fit of the data to the model was evaluated by the coefficient of determination, R^2^, by least-squares method[17].

### Biosorption isotherms

Equilibrium adsorption isotherms are usually used to determine the capacity, surface properties, and affinity of an adsorbent. Among all theoretical models, the Langmuir and Freundlich equilibrium models, the most widely used sorption isotherms, were chosen for the estimation of the adsorption capacity of *E. coli* E.

The linear Langmuir equation is written as follows[18]:


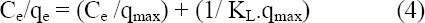


where q_e_ is the equilibrium biosorption capacity of biomass in nmol Cd (II)/mg of biomass, C_e_ is the equilibrium concentration of Cd (II) ion in nmol/L, q_max_ is the maximum amount of metal sorbed in nmol Cd (II)/mg of biomass, and K_L_ is the constant that is referred to the bonding energy of sorption in nmol/L. Langmuir isotherm refers to homogeneous adsorption, in which each molecule possesses constant enthalpies and sorption activation energy (all sites possess equal affinity for the adsorbate), with no transmigration of the adsorbate in the plane of the surface[19]. The linear Freundlich equation is written as follows[18]:


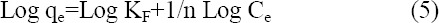


where q_e_ is the equilibrium biosorption capacity of the biomass in nmol Cd (II)/mg biomass, C_e_ is the equilibrium concentration of Cd (II) ion in nmol/L, and K_f_ (in nmol/L) and 1/n are constants related to the sorption capacity and intensity, respectively. Freundlich isotherm is the earliest known relationship describing the non-ideal and reversible adsorption, not restricted to the formation of monolayer. This empirical model can be applied to multilayer adsorption, with non-uniform distribution of adsorption heat and affinities over the heterogeneous surface[19].

Isotherm experiments were carried out at 30ºC, using 10 mg dried biomass/10 mL of varying initial Cd (II) concentrations in the range of 10–110 mg Cd (II)/L of Tris-HCl buffer (pH 6.5), with constant shaking at 100 rpm and using an equilibrium time of 1 h. The linear regression curve fitting procedure was performed as mentioned above.

### Response surface methodology (RSM)

One-factor-at-a-time, the classical method of experimental designs, involves changing one independent variable while maintaining all others at a fixed level. This method does not include the interactive effects among the variables. Experimental factorial designs can overcome this problem[20]. The RSM based on full or factorial design is a powerful tool for optimization, which have been employed extensively to optimize the biosorption of heavy metals[21-23].

In order to obtain the optimum conditions for Cd (II) adsorption, three variables, including pH, temperature, and biomass dosage of solution were selected for the study. The range for these factors was chosen based on preliminary screening experiments according to cell viability on LB agar. For this purpose, cell suspensions of the same weight were adjusted to different pH values using the following buffers (0.1 M): glycine-HCl (pH 3), sodium acetate (pH range of 4-5), Tris-HCl (pH range of 6-8), and glycine-NaOH (pH range of 9-10) and incubated at 30°C for 1 h with shaking at 100 rpm. In another test, cell suspensions of the same weight were incubated at different temperatures (20, 30, 40, 50, 60, 70, and 80ºC) for 1 h at pH 7 with shaking at 100 rpm. Subsequently, equal volumes of the cell suspensions were spread onto LB agar plates (pH 7) and incubated at 37 ºC overnight. Colony forming units were counted and compared to the condition with maximum colony forming units, at pH 7 and 30ºC.

The central composite design (CCD) based on RSM was used to optimize the above mentioned factors. There are three types of CCD, which depend on where the axial points are placed. In the face-centered CCD type, the axial points are at the center of each face of the factorial space, so α=±1. The high and low values of the factors are coded as +1 and -1, respectively. The mean value of the factors was assigned to 0 as the central point. The experimental design for the three mentioned factors contained a total of 17 experiments, representing six axial points on cubic surfaces and 2^3^ factorial points on vertices, as well as the central point with 3 replications (Table 1).

**Table 1:** Experimental design based on the central composite design used in this study

Trials	Point type	pH	Temprature (ºC)	Biomass dosage (mg/mL)
1	Factorial	5.0	20	10
2	Factorial	8.0	20	10
3	Factorial	5.0	40	10
4	Factorial	8.0	40	10
5	Factorial	5.0	20	30
6	Factorial	8.0	20	30
7	Factorial	5.0	40	30
8	Factorial	8.0	40	30
9	Axial	5.0	30	20
10	Axial	8.0	30	20
11	Axial	6.5	20	20
12	Axial	6.5	40	20
13	Axial	6.5	30	10
14	Axial	6.5	30	30
15	Central	6.5	30	20
16	Central	6.5	30	20
17	Central	6.5	30	20

The optimum values of the selected variables were obtained by solving the regression equation and also by analyzing the response surface plots using the Design Expert software (version 7.0.0; Stat-Ease, Inc., USA). The initial Cd (II) concentration was 40 mg/L, and each experiment was carried out in duplicate, with the results being reported as mean values±standard deviation.

### FTIR analysis

The FTIR study was intended to provide a deeper insight into the interaction between the surface functional groups of the biosorbent and the cadmium ions. The biomass was first dried prior and after adsorption using a lyophilizator (Denmark) and was grounded into fine particles using mortar and pestle. Each sample was then mixed with potassium bromide (1 mg in 100 mg of KBr), compressed into a 0.25-mm thickness disk and stabilized under controlled relative humidity before acquiring the spectrum. The FTIR spectrophotometer (Bruker-Vector22) used to record spectra was a Shimadzu IRPrestige in the wave number range of 400 to 4,000 cm^−1^.

### Desorption

Metals desorption is an important process in regeneration of the used adsorbents for their repeated use in water purification systems. It is also necessary to recover the precious metals. In this study, the binding sites of the bioadsorbent were first loaded with metal ions and desorption was then carried out to recover the adsorbed Cd (II). The cells were collected after adsorption under optimum conditions and washed with 0.1 M Tris-HCl. They were then incubated on ice for 15 min with 5 mM EDTA in 0.1M Tris-HCl (pH 8.0) to remove the surface-bound metals. Alternatively, following adsorption and washing, the cells were incubated with 0.1 M CaCl_2_ in 0.1 M Tris-HCl (pH 8.0) at 40ºC for 1 h. A control sample was also incubated in

Tris-HCl (pH 8.0) at 40ºC for 1 h. The supernatants resulting from these treatments were then subjected to atomic absorption analysis.

## RESULTS

### Kinetic studies

**Fig. 1:**
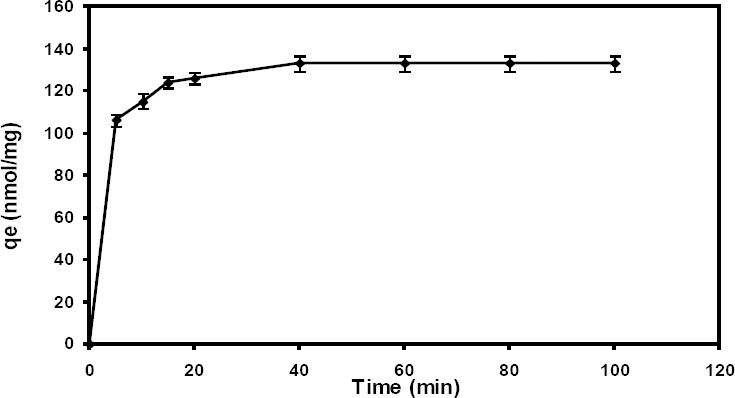
Effect of contact time on the biosorption capacity of Cd (II) (q_e_) by *E. coli* E. [Cd (II)] concentration, 20 mg/L; biomass dosage, 1 mg/mL Tris-HCl, pH 6.5, 30ºC, 100 rpm).

Figure 1 represents the effects of contact times (5 to 100 min) on the biosorption of Cd (II) by *E. coli* E. A fast rate of Cd (II) adsorption was observed in the first 5 min. The maximum removal of Cd (II) occurred after 40 min, when the uptake of Cd (II) was approximately 74.72%.

Biosorption kinetics was studied to understand the adsorption dynamics of Cd (II) onto the *E. coli* E surface. The models obtained in this study allowed to estimate the amount of Cd (II) adsorbed at the time of processing. Accordingly, two types of kinetic models were applied, the pseudo-first-order and pseudo-second-order.

The pseudo-first-order kinetic model is the plot of Log (qe–q_t_) versus time. Table 2 shows the values of the biosorption rate constant, K_1_, calculated q_e_, and experimental q_e_. The coefficient of determination (R^2^=0.75) showed that linear regression did not fit the experimental data (Table 2) and could not predict q_e_ accurately. Therefore, the pseudo-second-order kinetic model was used to analyze the biosorption kinetics of Cd (II). The pseudo-second-order kinetic model is the plot of t/q_t_ versus time. As shown in Table 2, according to the R^2^ of 0.99, the pseudo-second-order model could satisfactorily fit the experimental data, where the calculated q_e_ was in acceptable agreement with the experimental data.

**Table 2:** The biosorption rate constants and the q_e_ values from the pseudo-first-order and pseudo-second-order kinetics for the biosorption of Cd (II) by *E. coli* E

Metal ion	Expt. Q	Pseudo-first-order kinetics			Pseudo-second-order kinetics		
	
		R^2^	Cal. Q_e_ (nmol/mg)	K_1_ (1/min)	R^2^	Cal. Q_e_ (nmol/mg)	K_1_ (g/mg min)
Cd (II)	133 ± 3.53	0.75	16.34	0.035	0.99	135.13	0.005

Expt., experimental data of adsorbed metal; Cal., data of adsorbed metal calculated from the model

### Isotherms

**Fig. 2:**
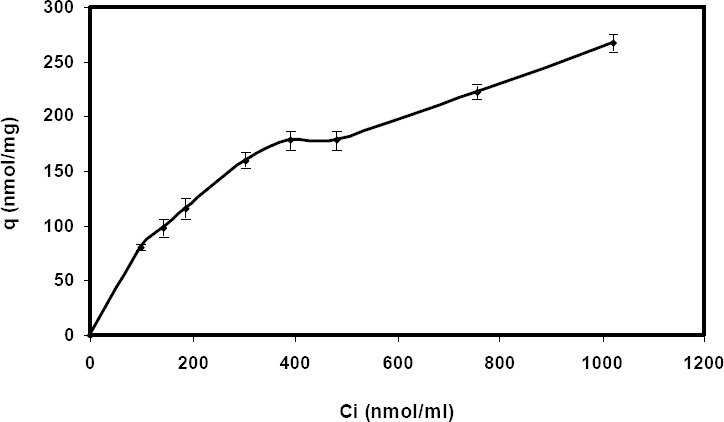
Biosorption of Cd (II) at various initial concentrations (pH 6.5, 1 mg/mL biomass, 30ºC).

The biosorption capacity of *E. coli* E was increased with the initial concentration of Cd (II) ions in solution (Fig. 2), demonstrating the potential of *E. coli* E, as a biosorbent, to treat wastewater containing high concentrations of metal ions.

The biosorption process will stop only when equilibrium is reached between the amount of metal ions adsorbed onto the biosorbent and that of solution. The isotherm models were thus investigated at the time of equilibrium. The parameters of equilibrium sorption were used to investigate the surface properties and affinity of the sorbent[24]. The Langmuir and Freundlich isotherm models were employed for this purpose.

The Langmuir adsorption model was applied for the experimental data obtained. A graph of C_e_/q_e_ versus C_e_ is a linear plot of the Langmuir model. The value of the biosorption capacity, q_max_, and the Langmuir constant, K_L_, were obtained from linear regression. The value of R^2^ for Cd (II) was found to be 0.97. Although the Langmuir model fits the experimental data for Cd (II), it was preferred to study the model fitting for the Freundlich model according to the R^2^ value of 0.97. The linear Freundlich plot can be obtained by the Log qe (nmol/mg) versus Log C_e_ (nmol/mL). According to R^2^ value of 0.98, the Freundlich isotherm was in good agreement with the experimental data in this study. The value of the 1/n was obtained as 0.32, indicating there is a chemical interaction between Cd (II) and *E. coli* E.

The adsorption constants estimated from the Freundlich and Langmuir isotherms are summarized in Table 3.

**Table 3:** Langmuir and Freundlich constants for Cd (II) biosorption by *E. coli* E

Langmuir			Freundlich		

K_L_	q_max_	R^2^	K_f_	1/n	R^2^
0.01	277.77	0.97	30.535	0.32	0.98

### Optimization of the biosorption process

The range of variables used in the optimization process was determined according to screening of variables based on cell viability. The *t*-test indicated a significant difference between pH values 4 and 7, as well as 9 and 7; however, no significant differences were found between pH values 5 and 7, 6 and 7, and 8 and 7 (*P*>0.05). Also, it demonstrated a significant difference between the temperatures 50, 60, 70, 80, and 30°C, but no significant difference was detected between temperatures 20, 30, and 40°C (*P*>0.05).

Consequently, the range of variables chosen to analyze the optimum conditions provided maximum biosorption efficiency using the face-centered CCD, as mentioned in Table 1. The Cd (II) uptake rate (q_e_) was measured as the response, and then the results were compared with the predicted values (Table 4). Analysis of variance (ANOVA) showed that there is a statistical significance in the quadratic model. The *F* and *P* values of the regression model were 91.42 and <0.0001, respectively, implying the significance of the model. R^2^ was 0.98, which indicates that only 0.28% of the total variable could not be explained by the model. The lack of fit value of 0.05 implied that the lack of fit is not significant relative to the pure error. Both parameters showed that the model could well fit the experimental data. The value of the adjusted R^2^ of 0.96 was in reasonable agreement with the predicted R^2^ of 0.93. In addition, a relatively low value of the coefficient of variation (CV=6.46) indicates the repeatability of the experiments.

**Table 4:** Observed and predicted values

Trial	Actual response q_e_ (nmol/ mg)	Predicted response
1	195.73±3.34	190.71
2	249.11±2.12	254.52
3	213.52±3.54	208.12
4	284.70±3.54	271.92
5	94.84±2.83	89.00
6	96.86±3.54	101.91
7	97.86±1.41	106.41
8	118.59±2.83	119.32
9	115.66±3.54	126.98
10	160.14±2.40	165.34
11	151.25±3.53	137.46
12	160.14±1.43	154.86
13	213.52±3.53	231.32
14	112.63±3.48	104.16
15	146.80 ±2.83	146.16
16	142.35±2.12	146.16
17	146.80 ±2.83	146.16

Eq indicates the final mathematical model corresponding to the coded factors after eliminating the insignificant terms (*P*>0.05), as determined by the Design-Expert software. (6):


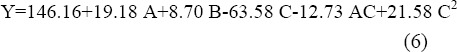


where Y shows q (metal uptake, nmol/mg); A, pH; B, temperature (ºC); C, biomass dosage (mg); AC, the interaction of pH and biomass dosage; C^2^, the second order effect of biomass dosage.

### Interactive effect of biomass dosage and pH of solution on biosorption

**Fig. 3:**
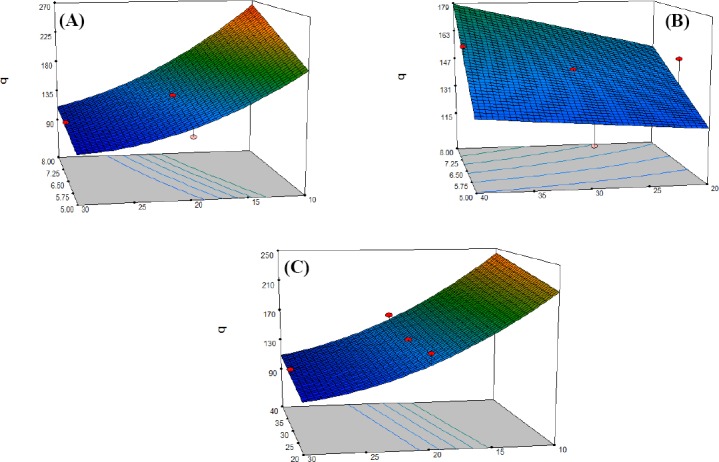
Response surface plot of different factors interactions. (A) The interactive effect of the biomass dosage and pH at constant temperature 30ºC on metal uptake (q); (B) the effect of temperature and pH at constant biomass dosage of 2 mg/mL on the metal uptake (q); (C) the effect of temperature and biomass dosage at constant pH of 6.5 on metal uptake (q).

Figure 3A shows the effect of the biomass dosage and pH of the solution on Cd (II) uptake. Our results indicated that metal uptake was decreased when the amount of biomass was increased from 1 to 3 mg/mL, and the pH was decreased from 8 to 5. Metal uptake, however, was at a maximum when the biomass concentration and pH value reached 1 mg/mL and of 8, respectively. Therefore, increased biomass dosages and decreased pH values simultaneously led to a reduction in the Cd (II) ion uptake.

### Interactive effect of temperature and pH on biosorption

Figure 3B represents the effect of temperature and pH in the solution on Cd (II) uptake. Our results demonstrated the decreased metal uptake after the temperature and pH were lowered from 40 to 20ºC and 8 to 5, respectively. The lowest uptake was observed at a temperature of 20ºC and pH value of 5.

### Interactive effect of biomass dosage and temperature on biosorption

The effect of biomass dosage and temperature on Cd (II) uptake is shown in Figure 3C. It was observed that the metal uptake was decreased when the amount of biomass was increased from 1 to 3 mg/mL, and the temperature was lowered from 40 to 20ºC, demonstrating the highest reduction in metal uptake at the biomass value of 1 mg/mL and a temperature of 40ºC.

Results from ANOVA showed that temperature has a significant effect on adsorption (*P*=0.0001). However, the *F* value of this factor (7.20) implied that this variable is not highly significant when compared with the biomass dosage (*F*=384.36). Hence, unlike the biomass dosage, a color range (blue to red) associated with temperature was not present in the response surface plot (Fig. 3C). A study, carried out by Hassan *et al*.[25] in 2009 showed that temperatures in a range from 25 to 55ºC have no remarkable effects on the copper biosorption by the brown seaweed *Sargassum* sp.

### Effect of the optimization of different variables

According to the results shown in Table 4, optimization of different variables led to increased cadmium adsorption from 94.84±2.83 to 284.70±3.54 nmol/ mg. This result was compared with the other biosorbents retrieved from selected literatures (Table 5).

**Table 5:** Comparison between cadmium adsorption of selected literatures and this work

Biosorbent	Operating condition				Amount adsorbed (nmol/mg)	Reference

	pH	Temp (ºC)	Biomass (g/L)	Time (h)		
*Bacillus circulan*	7	20	0.5	2	235.764	[37]
*Pseudomunas putida*	6	30	Not available	1	71	[38]
*Pseudomunas stutzeri*	5	30	1	30 min	387	[25]
*KCCM 34719*	5	28+2	1	>5	284.252	[27]
*Bacillus cereus RC-1*	Not	37	3.33	2	510
*Engineered E.coli*			available			
*Engineered E.coli*	8	40	0.01	1	284.697	this study

### FTIR analysis results

**Fig. 4:**
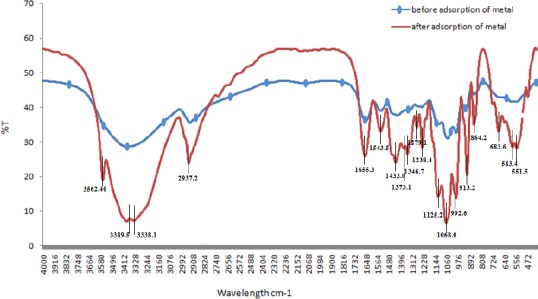
The FTIR spectra of our developed bioadsorbent in the absence and presence of cadmium.

FTIR analysis allows the identification of the functional groups involved in cadmium bioadsorption by shifting the changes in signal intensity compared to the control. FTIR analysis was carried out on our developed bioadsorbent (*E. coli* strain E) in the absence and presence of cadmium to determine the differences that are due to interaction of the metal ions with the surface functional groups (Fig. 4). Our results showed that cadmium ions bind to the functional groups, including carboxyl, amino, phosphoryl, thiol, and hydroxyl groups, which results in the shift and changes in the spectra of the bioadsorbent before and after cadmium adsorption.

The results attributable to the presence of the specific bands is summerized in Table 6. As shown in Figure 4, a weak –SH stretching was observed in the range of 2500-2600 cm^-1^. It has long been known that the SH stretch(s) mode, ν (SH), is generally found in the range of 2500-2600 cm^-1^, being weak in the IR[26]. The peaks at 2937.2, 1433, 1373 and 922 cm^−1^ might be due to CH_2_ stretching and bending vibrations, while the peaks at 922 and 913 cm^−1^ might be because of P-OR stretching. These groups could be attributed to polysaccharides, phospholipids, or ribose phosphate chain pyrophosphate, which are the main components of the bacterial cell wall[27]. The band observed at 1543.5 cm^−1^ was assigned to amide the bands of proteins and peptides.

**Table 6:** The FTIR Spectral Characteristics of Biosorbent before and after Biosorption of Cd

Wavelength range (cm^-1^)	Biosorbent after biosorption	Assignment	Functional class
3550-3600 3200-3550	3562.4	O–H	Oxime (=NOH)
3200-3550	3338.1	O–H or N–H	Alcohols, phenols or secondary amines
	3389.5	OO O–H or N–H	Alcohols, phenols or secondary amines
2500-3300	2937.2	C–H or O–H	Alkanes(methylene) or carboxylic acids
2600-2550	Very weak	S–H	Thiols
1630-1680	1655.3	C=C or C=N	Alkenes or oxime (=NOH)
1500-1560	1543.5	N=O or N–H	Nitro compounds or amides
1350-1470	1433.0	C–H or O–H	Alkanes and aldehyde or carboxylic acids
1330-1430	1373.1	C–H or O–H	Alkanes or alcohols, phenols
1325±25	1346.7	S=O	Sulfonic acid or sulfone
1210-1320	1279.1	C–O or N–O	Carboxylic acids or aromatic amine oxide
1210-1320	1238.4	C –O or P=O	Carboxylic acids or phosphonate and phosphoramide
1100-1200	1125.2	P=O	Phosphine oxide and phosphate
1050-1200	1068.0	C=S	Thiocarbonyl
880-1050	992.6	P–OR or =C–H & =CH_2_	Esters or alkenes
900-1050	913.2	P–OR (P–O–C)	(Phosphite) esters
600-900	864.2	C–H	Alkynes or arenes
600-900	683.6	C–H or O-H	cis-RCH=CHR alkynes and arenes or or NH_2_ and N-H alcohols, phenols or amines
500-700	583.4	C–Br and C–Cl	Bromoalkanes and chloroalkanes
500-600	551.5	C–Br and C–Cl	Bromoalkanes and chloroalkanes

### Desorption of Cd (II)

Because of the sensitivity of *E. coli* E to such conditions, no alkaline and acidic elutants were used for Cd (II) desorption. The recovery experiments showed that approximately 68.58% and 56.54% of the Cd (II) ions were recovered when 5 mM EDTA and 100 mM CaCl_2_ were used, respectively.

## DISCUSSION

In this study, the bioadsorbent developed in our previous study was evaluated by kinetic and isotherm studies, followed by optimization of adsorption conditions. Study on contact times showed that cadmium adsorption was initially rapid, presumably due to more active-binding sites present on the adsorbent in the beginning. However, the rate of cadmium adsorption became slower after some time, probably because of a lesser number of active-binding sites available on the adsorbent[28].

The first-order kinetic process was used for reversible reactions, in which equilibrium was established between liquid and solid phases. However, the pseudo-second order kinetic model assumes the chemical adsorption, as a probable rate-limiting step[29]. Bacterial cell walls contain several functional groups, including carboxyl, phosphonate, amine, and hydroxyl groups[30]. It is known that the functional groups present on the negatively-charged bacterial cell wall participate in the binding of metal cations, such as Cd (II)[30]. In addition, the cyanobacterial MT, SmtA, used in this adsorbent as a metal-binding component of this bioadsorbent contains a Zn_4_Cys_9_His_2_ cluster structure that can replace Cd (II) ions in the coordination bonds[31,32]. This result might be the main reason why the Cd (II) biosorption reaction by *E. coli* E has been well-modeled by the pseudo-second order kinetics. A number of studies have shown that the pseudo-second order mechanism is a better model for explaining the kinetics of divalent metal sorption onto heterogeneous sorbents[28,33].

According to Giles *et al*.[34], the plateau or the beginning of the linear portion above the “knee” must show “first-degree saturation” of the surface (Fig. 2); this observation conforms the conditions that all possible sites in the original surface are filled, and further adsorption would take place only on the new surfaces. For convenience, this degree of coverage may be called the formation of a complete “monolayer,” but this does not necessarily mean that it is a close-packed layer of single molecules or ions, as in a compressed monolayer on water[34]. Based on the R^2^ value, the experimental data was in good agreement with the Freundlich isotherm. It can be due to the presence of different functional groups on the *E. coli* E surface, especially, SmtA that produces a heterogeneous biosorbent. The Freundlich isotherm can fit various experimental adsorption data and has been found to be in line with data from the highly heterogeneous sorbent systems. For example, Gong *et al*.[35] have reported that the process of lead biosorption by *Spirulina maxima*, a filamentous cyanobacterium that can be used as a food supplement, follows the Freundlich isotherm model.

In optimization process, the interactive effect of biomass dosage and pH showed that increased biomass dosages and decreased pH values simultaneously lead to a reduction in Cd (II) ion uptake. This phenomenon can be attributed to cadmium precipitation at higher pH[36-38] or the reconstitution of SmtA molecules with cadmium ions, at higher pH values[32]. Masoudzadeh *et al*.[39] have stated that it might also be due to the fact that the available solute was insufficient to completely cover the available exchangeable sites on the cell surface, thereby resulting in low solute uptake at high biomass concentration. Similar observations have been made in study on Cd (II) biosorption using pretreated *Saccharomyces cerevisiae* biomass[22]. Meanwhile, biomass aggregation could interfere with the surface metal-binding sites. At high biomass dosages, the available binding sites are insufficient as they could be masked due to the surface protein interactions and limited metal accessibility[40]. It is likely that protons will then combine with the metal ions, thereby decreasing the interaction of the metal ions with the cell components[41].

The study of interaction between temperature and pH ranges on biosorption showed Cd (II) adsorption at a higher pH value. This finding can be related to the reaction between the binding sites of the sorbent and the metal, leading to the breaking of hydrogen bonds and the release of hydrogen ions, which are ultimately substituted by the metal ions[42,43]. In the process of physicosorption, weak adsorption interactions between surface and the metal ions was decreased with increased temperature[44]. However, based on the pseudo-second-order kinetic model, the interaction between *E. coli* E and Cd (II) ions can be ascribed to chemosorption. Therefore, temperature has a positive effect on the metal uptake capacity of *E. coli* E over the tested temperature ranges of 20-40ºC. Indeed, the results from this study, regarding the effect of temperature on Cd^2+^ adsroption, are consistent with those observed by Dang *et al*.[45]. On the other hand, the study of interaction between effects of biomass dosage and temperature on biosorption showed that although raising the biosorbent dosage caused an increase in the biosorbent surface area and the availability of more adsorption sites, the uptake capacity (q_e_) was decreased under such a condition. This result can be attributed to the unsaturated sites during the adsorption reaction, whereas the number of the sites available for the adsorption increases by raising the adsorbent dosage.

FTIR analysis was carried out to determine the functional groups involved in the cadmium bioadsorption. The significant changes in the appearance of new peak positions at specific wavelengths suggested that different compounds such as polysaccharides, phospholipids, proteins, and peptids were involved in the cadmium adsorption on the cell surface. A weak –SH stretching on FTIR spectra could be assigned to the molecules including thiol groups present in the cystein residues in MT on the surface of our developed bioadsorbent. According to the Hard-Soft Acid-Base theory, sulfur is a relatively soft (polarizable) atom. This also explains the tendency of thiol groups to bind to the soft elements/ions such as mercury, lead, or cadmium.

Our experiment regarding cadmium desorption using EDTA and CaCl_2_ showed that this kind of adsorption could be due to electrostatic and coordinance interactions occuring between Cd (II) ions and the *E. coli* E cell surface. The presence of competitor ions, such as calcium, at high concentrations, can release the Cd (II) ions involved in the electrostatic interactions. In addition, because of the affinity of heavy metals for EDTA, as a chelating agent, it could release the Cd (II) ions more efficiently than CaCl_2._ This result is consistent with that of Hua *et al*.[46] who indicated that the presence of EDTA generally decreased the adsorption of Cd to biofilm in natural waters.

Our results demonstrated that the presence of the different functional groups and MT present on the surface of the *E. coli* E caused an increased chemical adsorption on heterogenus surface. Furthermore, the surface adsorption was remarkabaly increased while the critical adsoprtion parameters were optimized. It can be concluded that surface engineering of the surface proteins involving metal adsorption as well as optimization of operational parameters have a great impact on the efficiency of biosorption process.

## References

[1] Boparai HK, Joseph M, O’Carroll DM (2013). Cadmium (Cd(2+)) removal by nano zerovalent iron:surface analysis, effects of solution chemistry and surface complexation modeling. Environmental science and pollution research international.

[2] Nouri J, Karbassi AR, Mirkia S (2008). Environmental management of coastal regions in the Caspian Sea. International journal of environmental science and technology.

[3] Torres E, Mera R, Herrero C, Abalde J (2014). Isotherm studies for the determination of Cd (II) ions removal capacity in living biomass of a microalga with high tolerance to cadmium toxicity. Environmental science and pollution research international.

[4] Dhir B (2014). Potential of biological materials for removing heavy metals from wastewater. Environmental science and pollution research.

[5] Klaassen CD, Liu J, Choudhuri S (1999). Metallothionein:an intracellular protein to protect against cadmium toxicity. Annual review of pharmacology and toxicology.

[6] Sigel A, Sigel H, Sigel RK (2009). Metallothioneins and related chelators:Metal Ions in Life Sciences.

[7] Bae W, Mehra RK, Mulchandani A, Chen W (2001). Genetic engineering of Escherichia coli for enhanced uptake and bioaccumulation of mercury. Applied and environmental microbiology.

[8] Pazirandeh M, Wells BM, Ryan RL (1998). Development of bacterium-based heavy metal biosorbents:enhanced uptake of cadmium and mercury by *Escherichia coli* expressing a metal binding motif. Applied and environmental microbiology.

[9] Sousa C, Kotrba P, Ruml T, Cebolla A, De Lorenzo V (1998). Metalloadsorption by *Escherichia coli* cells displaying yeast and mammalian metallothioneins anchored to the outer membrane protein lamb. Journal of bacteriology.

[10] Wu CH, Mulchandani A, Chen W (2008). Versatile microbial surface-display for environmental remediation and biofuels production. Trends in microbiology.

[11] Ghaedmohammadi S, Rigi G, Zadmard R, Ricca E, Ahmadian G (2015). Immobilization of bioactive protein A from *Staphylococcus aureus* (SpA) on the surface of *Bacillus subtilis* spores. Molecular biotechnology.

[12] Volesky B (2001). Detoxification of metal-bearing effluents:biosorption for the next century. Hydrometallurgy.

[13] Shi J, Lindsay WP, Huckle JW, Morby AP, Robinson NJ (1992). Cyanobacterial metallothionein gene expressed inEscherichia coli Metal-binding properties of the expressed protein. FEBS letters.

[14] Tafakori V, Ahmadian G, Amoozegar MA (2012). Surface display of bacterial metallothioneins and a chitin binding domain on *Escherichia coli* increase cadmium adsorption and cell immobilization. Applied biochemistry and biotechnology.

[15] Georgiou G, Stephens DL, Stathopoulos C, Poetschke HL, Mendenhall J, Earhart CF (1996). Display of β-lactamase on the *Escherichia coli* surface:outer membrane phenotypes conferred by Lpp–OmpA–β-lactamase fusions. Protein engineering.

[16] Vijayaraghavan K, Yun YS (2008). Bacterial biosorbents and biosorption. Biotechnology advances.

[17] Cruz CC, da Costa ACA, Henriques CA, Luna AS (2004). Kinetic modeling and equilibrium studies during cadmium biosorption by dead Sargassum sp. biomass. Bioresource technology.

[18] Bueno B, Torem M, Molina F, De Mesquita L (2008). Biosorption of lead (II), chromium (III) and copper (II) by R. opacus:Equilibrium and kinetic studies. Minerals engineering.

[19] Foo K, Hameed B (2010). Insights into the modeling of adsorption isotherm systems. Chemical engineering journal.

[20] Bezerra MA, Santelli RE, Oliveira EP, Villar LS, Escaleira LA (2008). Response surface methodology (RSM) as a tool for optimization in analytical chemistry. Talanta.

[21] Amini M, Younesi H, Bahramifar N, Lorestani AA, Ghorbani F, Daneshi A, Sharifzadeh M (2008). Application of response surface methodology for optimization of lead biosorption in an aqueous solution by *Aspergillus niger*. Journal of hazardous materials.

[22] Ghorbani F, Younesi H, Ghasempouri SM, Zinatizadeh AA, Amini M, Daneshi A (2008). Application of response surface methodology for optimization of cadmium biosorption in an aqueous solution by *Saccharomyces cerevisiae*. Chemical engineering journal.

[23] Rigi G, Mohammadi SG, Arjomand MR, Ahmadian G, Noghabi KA (2014). Optimization of extracellular truncated staphylococcal protein A expression in Escherichia coliBL21 (DE3). Biotechnology and applied biochemistry.

[24] Ho Y, Porter J, McKay G (2002). Equilibrium isotherm studies for the sorption of divalent metal ions onto peat:copper, nickel and lead single component systems. Water, air, and soil pollution.

[25] Hassan SH, Kim S-J, Jung A-Y, Joo JH, Eun Oh S, Yang JE (2009). Biosorptive capacity of Cd (II) and Cu (II) by lyophilized cells of Pseudomonas stutzeri. The Journal of general and applied microbiology.

[26] Qian W, Krimm S (1992). Conformation dependence of the SH and CS stretch frequencies of the cysteine residue. Biopolymers.

[27] Huang F, Dang Z, Guo C-L, Lu G-N, Gu RR, Liu H-J, Zhang H (2013). Biosorption of Cd (II) by live and dead cells of Bacillus cereus RC-1 isolated from cadmium-contaminated soil. Colloids and surfaces b:biointerfaces.

[28] Dubey A, Mishra A, Singhal S (2014). Application of dried plant biomass as novel low-cost adsorbent for removal of cadmium from aqueous solution. International journal of environmental science and technology.

[29] Srivastava VC, Swamy MM, Mall ID, Prasad B, Mishra IM (2006). Adsorptive removal of phenol by bagasse fly ash and activated carbon:equilibrium, kinetics and thermodynamics. Colloids and surfaces a:physicochemical and engineering aspects.

[30] Doyle RJ, Matthews TH, Streips UN (1980). Chemical basis for selectivity of metal ions by the Bacillus subtilis cell wall. Journal of bacteriology.

[31] Blindauer CA (2011). Bacterial metallothioneins:past, present, and questions for the future. Journal of biological inorganic chemistry.

[32] Blindauer CA, Harrison MD, Parkinson JA, Robinson AK, Cavet JS, Robinson NJ, Sadler PJ (2001). A metallothionein containing a zinc finger within a four-metal cluster protects a bacterium from zinc toxicity. Proceedings of the national academy of sciences of the united states of america.

[33] Reddad Z, Gerente C, Andres Y, Le Cloirec P (2002). Adsorption of several metal ions onto a low-cost biosorbent:kinetic and equilibrium studies. Environmental science and technology.

[34] Giles CH, MacEwan T, Nakhwa S, Smith D 786 Studies in adsorption. Part XI. A system of classification of solution adsorption isotherms, and its use in diagnosis of adsorption mechanisms and in measurement of specific surface areas of solids. Journal of the chemical society (resumed).

[35] Gong R, Ding Y, Liu H, Chen Q, Liu Z (2005). Lead biosorption and desorption by intact and pretreated Spirulina maxima biomass. Chemosphere.

[36] Puranik PR, Modak JM, Paknikar KM (1999). A comparative study of the mass transfer kinetics of metal biosorption by microbial biomass. Hydrometallurgy.

[37] Yilmaz EI, Ensari NY (2005). Cadmium biosorption by Bacillus circulans strain EB1. World journal of microbiology and biotechnology.

[38] Pardo R, Herguedas M, Barrado E, Vega M (2003). Biosorption of cadmium, copper, lead and zinc by inactive biomass of Pseudomonas Putida. Analytical and bioanalytical chemistry.

[39] Masoudzadeh N, Zakeri F, bagheri Lotfabad TB, Sharafi H, Masoomi F, Zahiri HS, Ahmadian G, Noghabi KA (2011). Biosorption of cadmium by *Brevundimonas sp* ZF12 strain, a novel biosorbent isolated from hot-spring waters in high background radiation areas. Journal of hazardous materials.

[40] de Rome L, Gadd GM (1987). Copper adsorption by *Rhizopus arrhizus* Cladosporium resinae and *Penicillium italicum*. Applied microbiology and biotechnology.

[41] Sağ Y, Kutsal T (1996). The selective biosorption of chromium (VI) and copper (II) ions from binary metal mixtures by R. arrhizus. Process biochemistry.

[42] Pagnanelli F, Esposito A, Toro L, Veglio F (2003). Metal speciation and pH effect on Pb, Cu, Zn and Cd biosorption onto Sphaerotilus natans:Langmuir-type empirical model. Water research.

[43] Tipping E (2002). Cation binding by humic substances.

[44] Zhou D, Zhang L, Zhou J, Guo S (2004). Cellulose/chitin beads for adsorption of heavy metals in aqueous solution. Water research.

[45] Dang VB, Doan HD, Dang-Vu T, Lohi A (2009). Equilibrium and kinetics of biosorption of cadmium(II) and copper(II) ions by wheat straw. Bioresource technology.

[46] Hua X, Hu J, Jiang X, Dong D, Guo Z, Liang D (2013). Adsorption of Cd to natural biofilms in the presence of EDTA:effect of pH, concentration, and component addition sequence. Environmental science and pollution research.

